# Contrast Enhanced Ultrasound Molecular Imaging of Spontaneous Chronic Inflammatory Bowel Disease in an Interleukin-2 Receptor α^−/−^ Transgenic Mouse Model Using Targeted Microbubbles

**DOI:** 10.3390/nano12020280

**Published:** 2022-01-17

**Authors:** Huaijun Wang, Jose G. Vilches-Moure, Thierry Bettinger, Samir Cherkaoui, Amelie Lutz, Ramasamy Paulmurugan

**Affiliations:** 1 Department of Radiology, School of Medicine, Stanford University, Palo Alto, CA 94304, USA; huaijun.morgan.wang@gmail.com (H.W.); alutz@stanford.edu (A.L.); 2 Department of Comparative Medicine, Stanford University, Stanford, CA 94305, USA; jvilches@stanford.edu; 3 Bracco Suisse SA, 1201 Geneva, Switzerland; thierry.bettinger@bracco.com (T.B.); samir.cherkaoui@bracco.com (S.C.)

**Keywords:** inflammatory bowel disease (IBD), colitis, ultrasound molecular imaging (USMI), transgenic mouse model, chronic disease, interleukin 2 receptor α deficient (IL-2Rα^−/−^) mice, P-selectin, E-selectin, remission, flare

## Abstract

Inflammatory bowel disease (IBD) is a lifelong inflammatory disorder with relapsing–remission cycles, which is currently diagnosed by clinical symptoms and signs, along with laboratory and imaging findings. However, such clinical findings are not parallel to the disease activity of IBD and are difficult to use in treatment monitoring. Therefore, non-invasive quantitative imaging tools are required for the multiple follow-up exams of IBD patients in order to monitor the disease activity and determine treatment regimens. In this study, we evaluated a dual P- and E-selectin-targeted microbubble (MB_Selectin_) in an interleukin-2 receptor α deficient (IL-2Rα^−/−^) spontaneous chronic IBD mouse model for assessing long-term anti-inflammatory effects with ultrasound molecular imaging (USMI). We used IL-2Rα^−/−^ (male and female on a C57BL/6 genetic background; *n* = 39) and C57BL/6 wild-type (negative control; *n* = 6) mice for the study. USMI of the proximal, middle, and distal colon was performed with MB_Selectin_ using a small animal scanner (Vevo 2100) up to six times in each IL-2Rα^−/−^ mouse between 6–30 weeks of age. USMI signals were compared between IL-2Rα^−/−^ vs. wild-type mice, and sexes in three colonic locations. Imaged colon segments were analyzed ex vivo for inflammatory changes on H&E-stained sections and for selectin expression by immunofluorescence staining. We successfully detected spontaneous chronic colitis in IL-2Rα^−/−^ mice between 6–30 weeks (onset at 6–14 weeks) compared to wild-type mice. Both male and female IL-2Rα^−/−^ mice were equally (*p* = 0.996) affected with the disease, and there was no significant (*p* > 0.05) difference in USMI signals of colitis between the proximal, middle, and distal colon. We observed the fluctuating USMI signals in IL-2Rα^−/−^ mice between 6–30 weeks, which might suggest a resemblance of the remission-flare pattern of human IBD. The ex vivo H&E and immunostaining further confirmed the inflammatory changes, and the high expression of P- and E-selectin in the colon. The results of this study highlight the IL-2Rα^−/−^ mice as a chronic colitis model and are suitable for the long-term assessment of treatment response using a dual P- and E-selectin-targeted USMI.

## 1. Introduction

Inflammatory bowel disease (IBD) is a chronic disease of the gastrointestinal tract. IBD comprises two distinct phenotypes: Crohn’s disease (CD), and ulcerative colitis (UC). The prevalence of IBD has been increasing during past decades, from developed to developing countries, especially in pediatric patients [1,2]. IBD is characterized as a life-long inflammatory disorder with relapsing-remission cycles. Acute flares of IBD occur in a random way and are usually unpredictable [3]. Currently, the diagnosis of IBD mainly relies on clinical symptoms and signs, along with laboratory and imaging findings. However, such clinical findings are not parallel to the disease activity of IBD [4,5], and they fail to provide clear indications for “go or no go” treatments, likely leading to either under- or over-treatments. Therefore, non-invasive, accurate, and quantitative tools are crucial for the multiple follow-up exams of IBD patients to monitor the disease activity and to determine treatment regimens.

Transabdominal ultrasound (US) imaging has been one of the first-line imaging tools along with CT and MRI for diagnosing IBD, especially in pediatric patients, because US has the advantages of no ionizing radiation as well as worldwide availability, high portability, excellent spatial resolution, and real-time imaging capability. However, sensitivity and specificity of standard US is low for quantitative evaluation of inflammation [6,7]. Contrast-enhanced ultrasound (CEUS) imaging using non-targeted contrast microbubbles (MBs) enables the evaluation of increased vasculature and changes in vascular dilation in the inflammatory bowel, and thus has been used in the assessment of treatment effects of IBD [8,9,10,11]. Since CEUS utilizes the first-pass of MBs for qualitative or quantitative analysis of perfusion parameters of the bowel, only one segment of the bowel can be imaged with one injection of MBs, which limits the use of CEUS in imaging acquisitions from multiple bowel segments per injection of MBs for evaluating the extent of disease in different locations of the bowels [12,13].

Ultrasound molecular imaging (USMI) using molecularly targeted contrast MBs is able to provide a quantitative readout of inflammation in IBD at the molecular level and to collect signals from multiple bowel segments after a single injection of MBs. The P- and E-selectin are two consistently expressed inflammation markers in patients of IBD [14,15,16]. These markers have been utilized as molecular imaging targets in dual P- and E-selectin targeted USMI. Dual P- and E-selectin targeted MBs (MB_Selectin_) can bind to P- and E-selectin overexpressed in the inflamed vascular endothelial cells upon systemic injection, and stay in the target vasculature for a prolonged period of time, allowing the imaging acquisitions of multiple bowel segments following a single administration of MB_Selectin_ [17,18]. Dual-selectin-targeted USMI has previously been used to differentiate different disease activities of inflammation and to assess treatment effects, and USMI findings were validated using standard references of ex vivo histology and P- and E-selectin expression in various rodent and swine models of IBD [17,19,20,21].

Mouse models of IBD have been extensively used for studying the mechanism of IBD during past decades. However, most of these models are chemically induced acute inflammation, which fail to recapitulate the chronic nature of IBD and thus are not ideal for evaluating long-term anti-inflammatory effects in weeks to months [17,19,21]. Genetically engineered models of IBD are preferred for studying spontaneous chronic IBD. However, there is a limited amount of studies where transgenic mouse models of IBD were used in USMI [18,22] that allowed for long term monitoring of chronic inflammation. Interleukin (IL)-2/IL-2 receptor (IL-2R) signaling pathway plays a critical role in the immune system [23,24,25]. It was reported that IL-2 (IL-2^−/−^) and IL-2Rα-deficient (IL-2Rα^−/−^) mice develop spontaneous colitis and systemic wasting disease [23,24,26,27]. Nonetheless, colitis in IL-2Rα^−/−^ mice was assessed solely with a traditional histology approach such as hematoxylin and eosin (H&E) staining. It is still unknown whether inflammatory changes in colon of IL-2Rα^−/−^ mice could be quantified using USMI with inflammation markers-targeted MBs in long-term follow-ups.

To the best of our knowledge, there has been no study where the spontaneous colitis in transgenic mice is evaluated using USMI at multiple time points over a longer period of time to determine the evolution of inflammation. Therefore, the purpose of this study was to evaluate a completely spontaneous chronic IBD in an IL-2Rα^−/−^ mouse model, whose lesions are easily accessible on USMI and resemble the phenotype of human IBD with remission-flare cycles, by using P- and E-selectin targeted USMI.

## 2. Materials and Methods

### 2.1. Synthesis of P- and E- Dual Selectin Targeted Microbubbles

Dual-selectin–targeted MBs (MB_Selectin_) that target both P- and E-selectin were synthesized by covalently coupling a truncated human selectin-binding glycoprotein ligand 1 fused to a human fragment crystallizable (Fc) domain (recombinant P-selectin glycoprotein ligand immunoglobulin G [rPSGL-Ig]), also known as YSPSL, provided by Y’s Therapeutics (San Bruno, CA, USA) onto lipid-shelled MBs as previously described [19,21,28]. Briefly, a fragmented version of the rPSGL-Ig ligand, binding to both P- and E-selectin, was attached to the MB’s wall to allow site-specific coup, ling of the ligand onto the MB shell. Firstly, rPSGL-Ig was treated with endoproteinase Lys-C (Thermo Scientific, Lausanne, Switzerland) at a 1/50 wt/vol ratio in TE buffer (25 mmol/L Tris HCl: 1 mmol/L EDTA (pH 8.5)). The mixture was incubated for 18 h at 37 °C to truncate a portion of the Fc domain of the rPSGL-Ig protein. After purification (performed by using the ANX Sepharose 4 fast-flow weak anion exchanger (GE Healthcare, Glattbrugg, Switzerland)), the rPSLG-Ig-fragment, still containing the active portion of the molecule, was isolated. This fragmented version of the rPSGL-Ig has only two lysyl residues per molecule, allowing site-specific conjugation of the ligand onto the MB shell at the side opposite to the active domain of the ligand. The MBs core consisted of a mixture of perfluorobutane (C_4_F_10_) and nitrogen (N_2_), and the lipid shell was composed of a mixture of 1,2-distearoyl-*sn*-glycero-3-phosphocholine (DSPC; Genzyme Pharmaceutical, Liestal, Switzerland), palmitic acid (Fluka, Buchs, Switzerland), and 1,2-distearoyl-*sn*-glycero-3-phosphoethanolamine-*N*-[maleimide (polyethylene glycol)-2000] (DSPE-PEG2000-maleimide; Avanti Polar Lipids, Alabaster, Ala). MBs were lyophilized and stored in septum-sealed vials with a 35/65 vol/vol mixture of perfluorobutane and nitrogen. The reconstituted bubbles in physiological saline was used for animal experiments.

### 2.2. Animals

**Figure 1:**
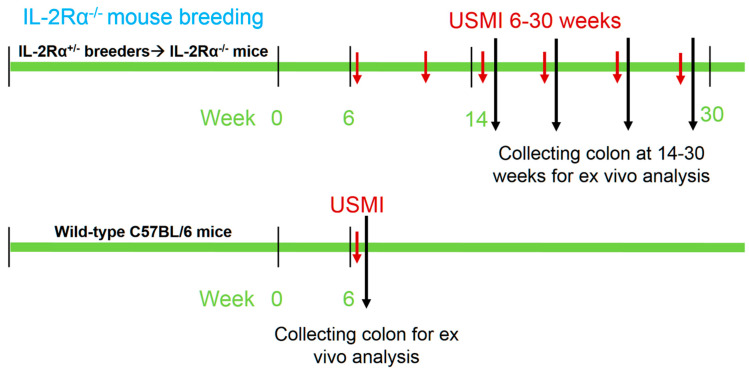
Overall experimental design.

The institutional Administrative Panel on Laboratory Animal Care approved all procedures involving laboratory animals. Commercially available heterozygous male and female mice of IL-2Rα^+/−^ (B6.129S4-*Il2ratm1Dw*; stock No: 002952) on a C57BL/6J background were initially purchased from The Jackson Laboratory (Bar Harbor, ME) as breeding pairs. The IL-2Rα^+/−^ mice were bred in the institution. All the mice were maintained in individually ventilated cages, fed with sterile commercial rodent chow, and provided sterile drinking water ad libitum. Mouse genotypes of their offspring were determined from tail biopsies using real time polymerase chain reaction (qPCR) with specific probes designed for IL-2Rα gene (Transnetyx, Cordova, TN, USA). IL-2Rα^−/−^ mice (male *n* = 21; female *n* = 18; total *n* = 39) between 6–30 weeks of age were used for USMI in this study. The experimental design is summarized in Figure 1, including the mouse breeding, serial in vivo USMI exams, and ex vivo analysis including histology and immunofluorescence staining for P- and E-selectin.

### 2.3. Ultrasound Molecular Imaging

We used a dedicated Vevo 2100 (VisualSonics, Toronto, ON, Canada) small animal ultrasound scanner with a dedicated small animal transducer (MS250; center frequency of 18 MHz; lateral and axial resolution of 165 μm and 75 μm, respectively; transmit power, 10%; mechanical index, 0.2; dynamic range, 35 dB) for the USMI study. We used a clinically compatible MB_Selectin_ (Bracco Suisse SA, Geneva, Switzerland) microbubble for the USMI study. The formulation of MB_Selectin_ was followed as described previously [21].

Mice were positioned on the heated US scanning table under gas anesthesia with 2% isoflurane in 2 L room air/min. Since the inflammatory lesions may not be continuous along the colon, we selected three locations of descending colon in each mouse sequentially for imaging following the single injection of MBs [18]. First, the proximal (2.5 cm from anus), middle (2.0 cm from anus) and distal (1.5 cm from anus) locations of descending colon (so called proximal, middle, and distal colon onwards) were localized in B-mode using an electronic caliper available on the US scanning table and marked on the skin of abdomen using a Sharpie marker. Afterwards, imaging was switched to contrast mode, and the transducer was placed on the proximal colon first. MB_Selectin_ of 5 × 10^7^ in 100 µL saline solution was manually injected via tail vein or retro-orbital sinus using a catheter over 2 s. At 4 min after injection of MBs, images were acquired for the proximal colon. 10 s of axial imaging frames were first acquired for USMI signals (pre-destructive) from both freely circulating MBs and MBs molecularly bound to the selectin receptors of the vasculature in the field of view (FOV). Then, destruction pulses (transmit power, 100%; mechanical index, 0.63) were applied for one second to destroy all circulating and bound MBs in the FOV. This was followed by the acquisition of 10 s of imaging frames for USMI signals (post-destructive) from freely circulating MBs only. Following imaging acquisition from the proximal colon, the transducer was immediately moved to the middle and distal colon sequentially, and USMI data sets were acquired respectively using the aforementioned imaging protocol. Each imaging session contained three imaging acquisitions from proximal, middle, and distal colon, respectively, and lasted for 5.5 min.

All the IL-2Rα^−/−^ mice (male *n* = 21; female *n* = 18; total *n* = 39) were imaged between 6 to 30 weeks. Each mouse was imaged up to 6 times at different time points, and the time points and frequency for imaging acquisition per mouse were randomly chosen to obtain the evolution of USMI signals between 6 to 30 weeks. In addition, a group of wild-type mice (male *n* = 3; female *n* = 3; total *n* = 6) were imaged once serving as negative control without colitis.

### 2.4. Analysis of Ultrasound Molecular Imaging Data

We analyzed all the data sets in a random order with commercially available software (Vevo Lab; VisualSonics, Toronto, ON, Canada) by a reader with 9 years of experience in analyzing ultrasound images. On all the images from one imaging acquisition, a region of interest (ROI) was manually drawn covering the colon wall. We quantified the level of USMI signal from the attached microbubbles by measuring the difference between the imaging signal intensity within the ROI pre- and post-destructive data using an arbitrary unit (a.u.).

According to the USMI signals in the negative control mice (0–0.9 a.u.), the USMI signals of active colitis were assigned to be ≥1.0 a.u. Thus, the USMI signals <1.0 a.u. were deemed to represent the absence of active colitis; signals of 1.0–2.4 a.u.: low USMI signal intensity; signals of 2.5–4.9 a.u.: medium USMI signal intensity; signals ≥5.0 a.u.: high USMI signal intensity. When the signal of one imaging acquisition was ≥1.0 a.u., that imaging acquisition was determined to represent active colitis. When a mouse had at least one positive imaging acquisition, that mouse was assigned to be positive for active colitis.

### 2.5. Ex Vivo Analysis of Colon Tissues

Ex vivo analysis of colonic tissues was performed between 14–30 weeks. The mice with background, low, medium, and high USMI signals were randomly chosen for ex vivo analysis to correlate between in vivo USMI signals and ex vivo P- and E-selectin expression. We harvested colon tissues respective to the imaging locations at the end of the study after sacrificing the animals.

For immunofluorescence staining, we fixed the tissues in 4% paraformaldehyde (PFA) at 4 °C overnight, and then cryopreserved them in a 30% sucrose solution. We then placed the samples in optimal cutting temperature (OCT) solution, and then froze them by keeping in dry ice. From each tissue sample, 10 µm sections were obtained by using a cryomicrotome. Sections were incubated in phosphate buffered saline (PBS) for 10 min to remove the OCT, and permeabilized for 10 min in PBS solution with 0.5% Triton-X 100. Sections were blocked in a 3% bovine serum albumin solution (Sigma; St. Louis, MO, USA) containing 3% goat serum (Sigma) and 3% donkey serum (Sigma) for 30 min at room temperature prior to incubation with primary antibodies [rabbit anti-mouse P-selectin (Abcam, Cambridge, MA, USA) or rabbit anti-mouse E-selectin (Abcam) and rat anti-mouse CD31 (BD Biosciences, San Jose, CA, UAS)]. Primary antibodies were made visible with secondary antibodies of AlexaFluor488 donkey anti-rat IgG (Invitrogen, Grand Island, NY, USA) and AlexaFluor546 goat anti-rabbit IgG (Invitrogen). Samples were mounted in aqueous mounting media (BiogeneX, San Ramon, CA, USA) and imaged using a 20× objective on a Leica TCS SP8 metaconfocal microscope (Leica Microsystems Inc., Buffalo Grove, IL, USA). Single confocal slices were collected and displayed.

For H&E staining, we collected colonic tissues and fixed in 4% paraformaldehyde (PFA), The tissues were sectioned into 5 μm-thick slices and stained using H&E according to a standard protocol [29]. We examined the sections using an upright brightfield microscope (Olympus BX43; Tokyo, Japan). We acquired H&E images using an Olympus DP27 camera with cellSens software. We examined the tissues for evidence of fibrosis, edema, ulceration, and inflammation. We defined fibrosis as the deposition of eosinophilic collagenous matrix leading to separation of the colonic gland profiles. Similarly, the edema was defined as expansion of the tissue compartments by clear spaces or pale eosinophilic homogeneous glassy material. Ulceration was defined as an epithelial defect in the mucosal lining. Inflammation was defined as the presence of inflammatory cells scattered throughout the lamina propria, leading to the separation of colonic glandular profiles. Discrete lymphofollicular aggregates (generally attributed to antigenic stimulation) were not considered as true inflammatory foci.

### 2.6. Statistical Analysis

All continuous measurements were expressed as mean ± standard deviations. We used the paired Wilcoxon rank test to compare USMI signals with MB_Selectin_ of the same location (proximal, middle, or distal colon) between different time points and to compare the USMI signals of the same mice between proximal, middle, and distal colon, respectively, whenever the data were paired. Similarly, the two-sample Wilcoxon rank test was used to compare USMI signals of imaging acquisitions between IL-2Rα^−/−^ and wild-type mice, to compare the USMI signals between male and female IL-2Rα^−/−^ mice, and to compare USMI signals of the mice between different time points, when the data were not paired. All statistical analyses were performed with statistical software (SPSS version 21; IBM Corporation, Endicott, NY, USA). The significance level was set as anything less than 0.05.

## 3. Results

### 3.1. General Conditions

Homozygous IL-2Rα^−/−^, heterozygous IL-2Rα^+/−^ and wild-type (IL-2Rα^+/+^ littermates) mice were obtained with expected Mendelian frequency. IL-2Rα^−/−^ mice before weaning were healthy and not visually distinguishable from wild-type littermates. After weaning, IL-2Rα^−/−^ mice started to present signs of autoimmune disorders, such as anemia. Approximately 4.9% (2/41; not recruited for USMI) of IL-2Rα^−/−^mice died before 6 weeks of age when the USMI was started; 26.8% (11/41) of mice died at the age of between 6–30 weeks of the USMI window. Signs of autoimmunity were exacerbated with age in most IL-2Rα^−/−^ mice. All the IL-2Rα^−/−^ mice were sacrificed by week 30, following the institutional animal care policy.

### 3.2. In Vivo Ultrasound Molecular Imaging

#### 3.2.1. Spontaneous Colitis in IL-2Rα^−/−^ Mice with 100% Penetrance

**Figure 2:**
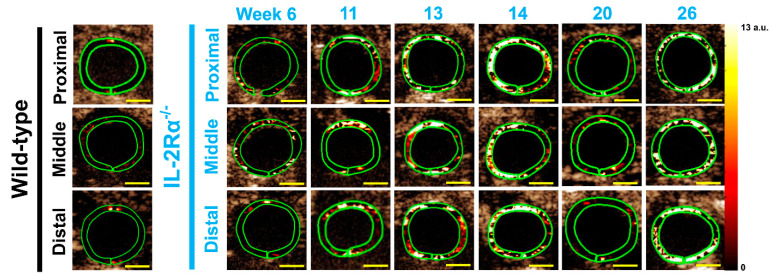
Axial dual P- and E-selectin targeted ultrasound molecular images of proximal, middle, and distal colon in a representative IL-2Rα^−/−^ mouse from week 6 to 26 and in a representative wild-type mouse at 6 weeks of age. Compared to the wild-type mouse, the IL-2Rα^−/−^ mouse showed substantially higher imaging signals from week 6 to 26, and the imaging signals fluctuated with being higher at week 14 and 26 than other time points. The imaging signals did not show a substantial locational difference between proximal (**top row**), middle (**middle row**) and distal colon (**bottom row**). Scale bars, 1 mm.

Thirty-nine IL-2Rα^−/−^mice (male = 21; female = 18) underwent USMI between 6–30 weeks. In each imaging session, three imaging acquisitions (proximal, middle, and distal colon) were performed in each mouse. Each mouse was imaged at up to 6 time points between 6–30 weeks (Figure 2). Therefore, there were 387 imaging acquisitions (male = 201; female = 186) from all the IL-2Rα^−/−^ mice (Table 1). Six wild-type mice (male = 3; female = 3) were imaged once and sacrificed for ex vivo analysis as negative controls. 42.9% (166/387) of imaging acquisitions in the IL-2Rα^−/−^ mice were positive for active colitis, and 100% (39/39) of mice were positive for active colitis (Table 1). During the first six weeks of age, we closely monitored the progression of the mice for early mortality and did not perform USMI. We started to image the mice at 6 weeks of age. The earliest onset of colitis was observed at 6 weeks, and the latest at 14 weeks (mean onset age, 10.5 ± 2.4 weeks). IL-2Rα^−/−^ mice developed spontaneous colitis with 100% penetrance between 6–14 weeks, which could be detected on dual selectin-targeted USMI (Table 1).

#### 3.2.2. Spontaneous Colitis in IL-2Rα^−/−^ Mice without Sex or Location Difference

**Figure 3:**
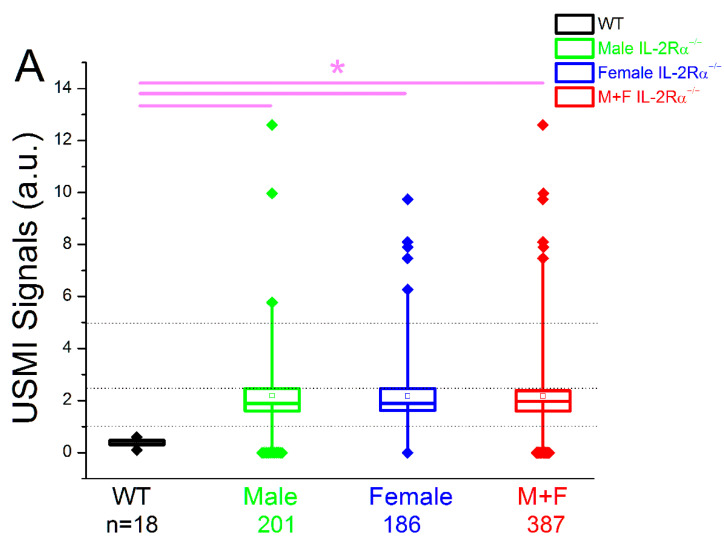

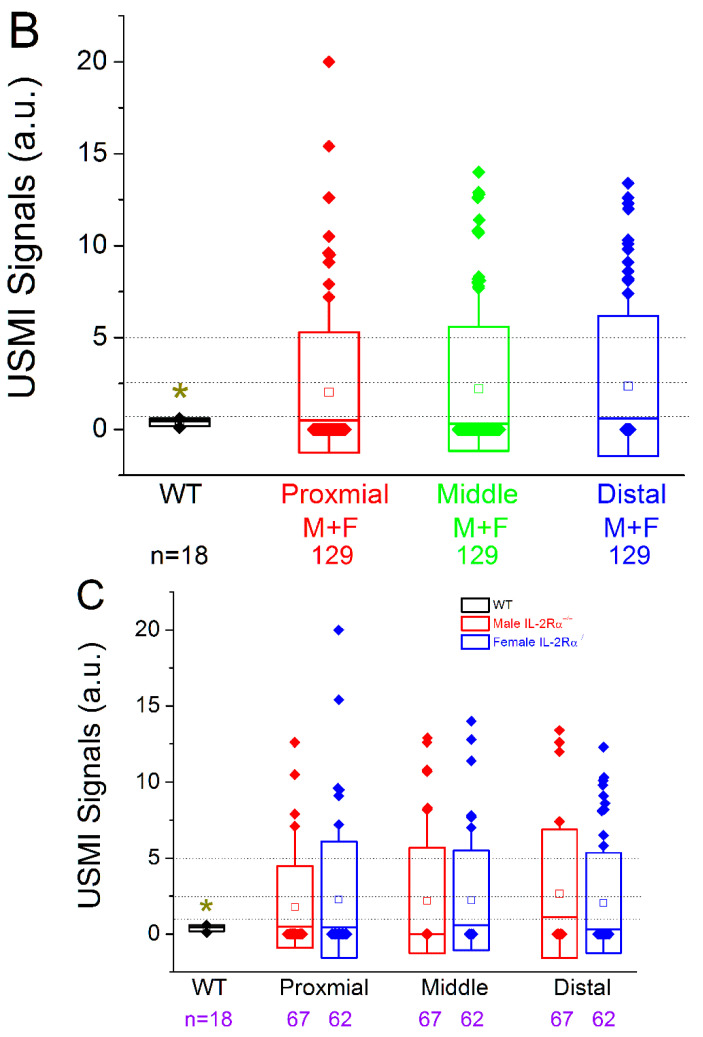
IL-2Rα^−/−^ mice showed spontaneous colitis without sex or locational difference between 6–30 weeks of age. (**A**) The mean USMI signals of all the imaging acquisitions from proximal, middle and distal colon in both male and female IL-2Rα^−/−^ mice were significantly higher than wild-type mice, and there was no significant difference in USMI signals between male and female IL-2Rα^−/−^ mice. (**B**) When the imaging acquisitions were pooled according to proximal, middle and distal colon in both male and female IL-2Rα^−/−^ mice, the mean USMI signals of proximal, middle, and distal colon were significantly higher than wild-type mice, and there was no significant difference in USMI signals observed between proximal, middle and distal colon in both male and female IL-2Rα^−/−^ mice. (**C**) When USMI signals of proximal, middle, and distal colon were further divided according to male and female IL-2Rα^−/−^ mice, mean USMI signals of proximal, middle, or distal colon in either male or female IL-2Rα^−/−^ mice were significantly higher than wild-type mice, and did not show a significant difference in USMI signals between the 3 locations or between the sexes. Each box in the plot represents the 25th and 75th quartiles while the line inside the box identifies the median. The small square within the box indicates the mean. ♦ represents outliers; * indicates *p*-value < 0.05 between the two boxes under the bar (**A**) or between the box of wild-type mice and any box in IL-2Rα^−/−^ mice (**B,C**).

First, the USMI signals from three imaging acquisitions of the proximal, middle, and distal colon in the same imaging session of an IL-2Rα^−/−^ mouse were averaged out as a single value of imaging signal per mouse per time point. Then, all the single values from all the IL-2Rα^−/−^ mice imaged at multiple time points between 6–30 weeks were pooled to obtain a single value for comparison. The mean value of USMI signals of 2.2 ± 2.3 a.u. in IL-2Rα^−/−^ mice was significantly (*p* < 0.001) higher than wild-type mice (0.4 ± 0.2 a.u.). The USMI signals in either male (2.2 ± 2.4 a.u, *p* < 0.001) or female (2.2 ± 2.3 a.u, *p* < 0.001) IL-2Rα^−/−^ mice were significantly higher than wild-type mice, but there was no significant difference (*p* = 0.996) between male and female IL-2Rα^−/−^ mice (Figure 3A).

When all the imaging acquisitions were pooled according to locations of proximal, middle, or distal colon in both male and female IL-2Rα^−/−^ mice, the mean values of the USMI signals of proximal (2.0 ± 3.3 a.u, *p* < 0.001), middle (2.2 ± 3.4 a.u, *p* < 0.001), and distal (2.4 ± 3.8 a.u, *p* < 0.001) colon were significantly higher than wild-type mice (0.4 ± 0.2 a.u.), respectively (Figure 3B). In addition, mean values of USMI signals did not show any locational difference between proximal, middle, and distal location (*p* = 0.62 for proximal vs. middle; *p* = 0.39 for proximal vs. distal; *p* = 0.71 for middle vs. distal colon) (Figure 3B). Also, the mean value of USMI signals of each location (proximal/middle/distal colon) in either male or female IL-2Rα^−/−^ mice, respectively, was significantly higher than wild-type mice (all *p* < 0.001), and did not show a significant difference between sex (male/female) or location (proximal/middle/distal colon) (all *p* > 0.05) (Figure 3C). These data indicate the overall spontaneous inflammatory status of colon as shown on dual selectin-targeted USMI in IL-2Rα^−/−^ mice between 6–30 weeks, and the USMI signals of colitis did not show any significant difference between male and female IL-2Rα^−/−^ mice, or significant locational difference between proximal, middle, and distal colon.

### 3.3. Remission Flare-Like Pattern of Spontaneous Chronic Colitis in IL-2Rα^−/−^ Mice

**Figure 4:**
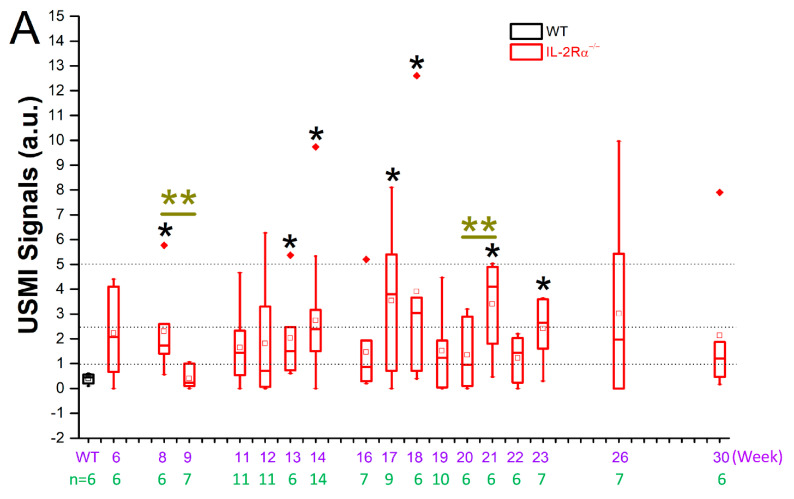

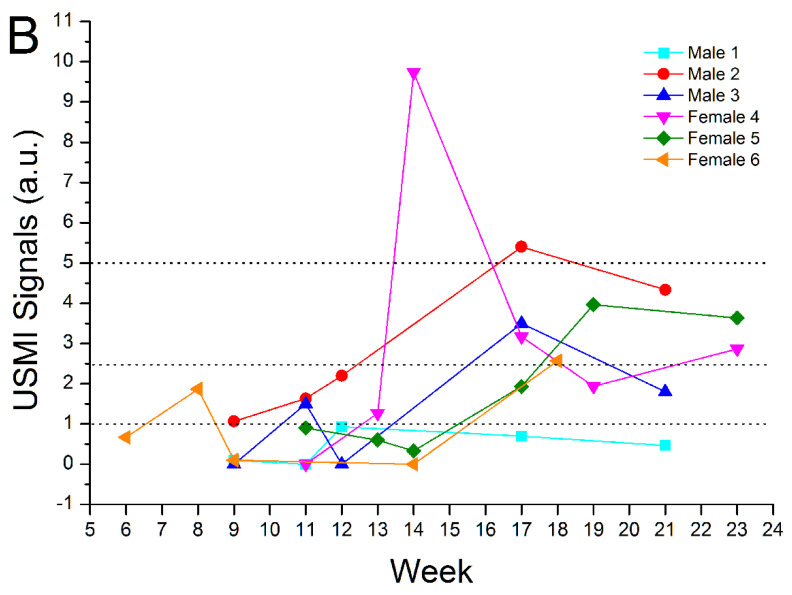
USMI signals fluctuated between 6–30 weeks of age in IL-2Rα^−/−^ mice. First, the USMI signals from three USMI imaging acquisitions of the proximal, middle, and distal colon in an IL-2Rα^−/−^ mouse in the same imaging session were averaged out as a single value of imaging signal per mouse per time point. Afterwards, all the single values from all the IL-2Rα^−/−^ mice imaged at multiple time points between 6–30 weeks were pooled together. (**A**) The mean USMI signals in IL-2Rα^−/−^ mice between 6–30 weeks were higher than wild-type mice, and fluctuated between the 2 consecutive time points. Black * indicates *p* value < 0.05 between the box of wild-type mice and the box of IL-2Rα^−/−^ mice; olive ** indicate *p* value < 0.05 between the two boxes under the bar. (**B**) The evolution of inflammation degree at multiple time points from the six representative IL-2Rα^−/−^ mice. Most of the UMSI signals were higher than 1.0 a.u., while some of them fell below 1.0 a.u. There was at least one peak of USMI signals in each mouse, suggesting the recapitulation of remission-flare pattern in patients with IBD. The frequencies and duration of remission-flare vary in each mouse.

USMI signals from three imaging acquisitions of the proximal, middle, and distal colon in the same imaging session of an IL-2Rα^−/−^ mouse were averaged out as a single value of imaging signal per mouse per time point. Mean USMI signals of all the single values in IL-2Rα^−/−^ mice between 6–30 weeks were significantly higher than wild-type mice. In contrast, the USMI signals in IL-2Rα^−/−^ mice showed fluctuation in the imaging signal between weeks 6–30 (Figure 2 and Figure 4A). USMI signals at weeks 8, 13, 14, 17, 21, and 23 were significantly (all *p* < 0.05) higher than wild-type mice. When the USMI signals were compared between the two consecutive time points, the USMI signals at week 9 were significantly lower than at week 8, and the USMI signals at week 21 were significantly higher than at week 20 (both *p* < 0.05) (Figure 4A).

For individual mice, averaged USMI signals from the proximal, middle, and distal colon fluctuated above and below 1.0 a.u. between 6–30 weeks. Figure 4B shows the evolution of USMI signals in 6 selected IL-2Rα^−/−^ mice (male: *n* = 3; female: *n* = 3) between 6–23 weeks. In No.1 mouse, the fluctuating USMI signals were mostly lower than 1.0 a.u; in No.2 mouse, the fluctuating USMI signals were always higher than 1.0 a.u.; in No.3–6 mice, the fluctuating USMI signals were either higher or lower than 1.0 a.u.. The fluctuating USMI signals in the IL-2Rα^−/−^ mice between weeks 6–30 might suggest the recapitulation of remission-flare pattern in patients with IBD, while there was no clear trend observed in how the USMI signals evolved, e.g., the durations and frequencies of remission or flare phases (Figure 4B).

### 3.4. Ex Vivo Analyses

#### 3.4.1. Hematoxylin and Eosin (H&E) Staining

**Figure 5:**
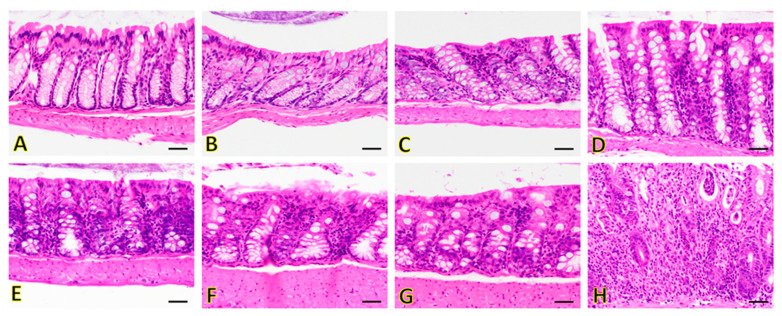
Hematoxylin and eosin (H&E) stained sections of colon in representative IL-2Rα^−/−^ mice and wild-type mice. Wild-type (**A**,**B**) mice show no evidence of inflammation in the colon. Inflammation in the colon increases over time in IL-2Rα^−/−^ mice. At 14 weeks (when all the IL-2Rα^−/−^ mice had developed colitis), the colon of IL-2Rα^−/−^ animals shows minimal inflammatory cell infiltration expanding the lamina propria (**C**). Inflammatory cell infiltrate generally increases over time in IL-2Rα^−/−^ animals from 18 weeks (**D**), 23 weeks (**E**), 26 weeks (**F**), to 30 weeks (**G**,**H**). Edema, fibrosis, and ulceration were not observed, and neutrophilic inflammation was only observed at 30 weeks (**G**,**H**). Magnification: 40×. Scale bar: 20 μm.

The colon tissues were categorized into IL-2Rα^−/−^ (tissues collected 14–30 weeks of age), and wild-type cohorts (Figure 5). When present, changes were generally mild (except for late time points). In the colon of IL-2Rα^−/−^ mice, inflammation was the main change observed, and was usually present as scattered foci (and not diffuse throughout the tissue). Non-inflammatory discrete lymphofollicular were not considered true inflammatory foci and were present in all animals (both IL-2Rα^−/−^ and controls). At earlier time points (14 weeks), the colon of IL-2Rα^−/−^ animals had minimal inflammatory cell infiltration expanding the lamina propria (Figure 5C) compared to wild-type animals (which were devoid of inflammatory infiltrate; Figure 5A,B). Inflammatory cell infiltrate generally increased over time in IL-2Rα^−/−^ animals (Figure 5D–H). At later time points (14–30 weeks), the degree of inflammatory cell infiltrate was variable, ranging from minimal and similar to that seen at earlier time points with scattered inflammatory cells minimally extending the lamina propria (Figure 5G) to prominent inflammation that separated the crypts and spilled into the crypt lumen (Figure 5H). Edema, fibrosis, and ulceration were not observed, and neutrophilic inflammation was only observed at later time points (Figure 5H).

#### 3.4.2. Immunofluorescence Staining

**Figure 6:**
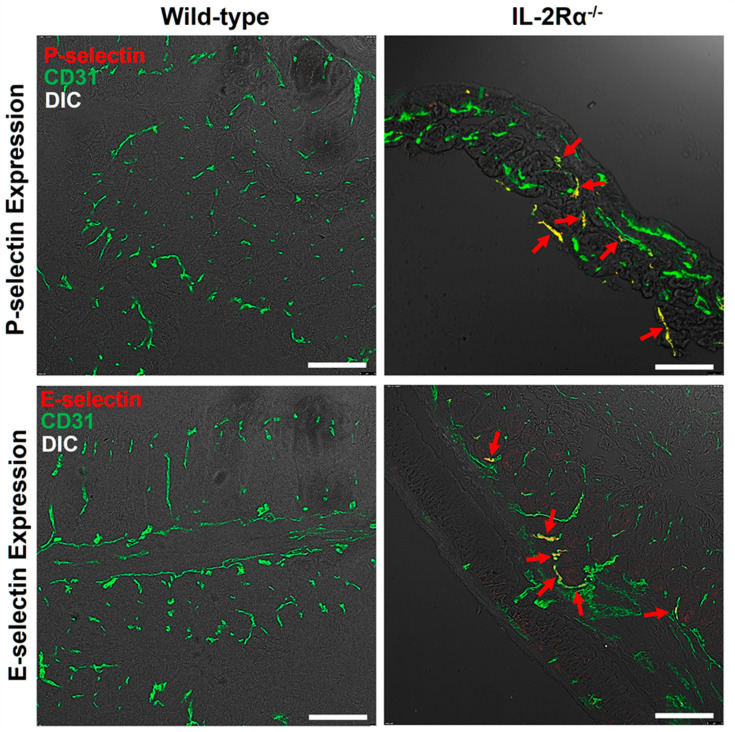
P- and E-selectin expression of colon from representative IL-2Rα^−/−^ and wild-type mice. Representative wild-type mice (**left column**) showed background expression of P-selectin (**top row**) and E-selectin (**bottom row**) in the colon. Representative IL-2Rα^−/−^ mice (**right column**) demonstrated strong expression (**red arrows**) of P-selectin (**top row**; highlighted in red) and E-selectin (**bottom row**; highlighted in red) on CD31-positive vascular endothelial cells (highlighted in green) in the inflamed colon. Confocal micrographs were overlaid on differential interference contrast (DIC) images. Scale bars, 100 μm.

P- and E-selectin expression was assessed in the IL-2Rα^−/−^ mice between 14–30 weeks and wild-type mice at 6 weeks (Figure 6)**.** The P- and E-selectin expression level correlated well with the in vivo imaging signal intensities. There was only background expression of P- and E-selectin was observed in wild-type mice (Figure 6). Mild, moderate to strong expression of P- and E-selectin was observed in the IL-2Rα^−/−^ mice with active colitis (USMI signals ≥1.0 a.u.) in the imaging session prior to tissue collection (Figure 6).

## 4. Discussion

This study shows that IL-2Rα^−/−^ mice develop spontaneous chronic colitis, which can be detected using P- and E-selectin-targeted USMI starting at 6 weeks of age without substantial difference between male and female mice or between the locations of the colon. The result also showed a prominent correlation between USMI findings and ex vivo histology and immunofluorescence staining. In addition, we observed a fluctuating pattern of USMI signals that may recapitulate the remission-flare pattern of human IBD. Therefore, the spontaneous chronic model of colitis in IL-2Rα^−/−^ mice can be used for long-term repeated monitoring of the disease, and for longitudinal assessment of anti-inflammatory treatment response using dual-selectin targeted USMI.

Various mouse models of IBD have been used in quantifying inflammation using USMI [30]. Human IBD is characteristic of a natural occurrence and chronic course—often lifelong—with remission-flare phases. Transgenic mouse models of chronic IBD are appealing for evaluation of evolving inflammation with USMI due to advantages of spontaneous onset, chronicity, resemblance to human IBD and subsequent clinical relevance [18,22]. However, there are limitations of these models. For instance, it takes more effort to develop spontaneous IBD models in transgenic mice. To the best of our knowledge, there are only two studies where IBD in transgenic mice has been evaluated using USMI. In a study by Bachmann et al., ileitis (SMAP model) was imaged using USMI with mucosal addressin cellular adhesion molecule (MAdCAM)-1 targeted MBs [22], but the ileum is not ideal for repeated ultrasound imaging due to relative motility of this segment. In another study by Wang et al., IL-10^−/−^ transgenic mice were evaluated with USMI; these animals failed to develop spontaneous colitis, and colitis was only sustained when inflammation-inducing drugs/chemicals were continuously administered [18].

In the current study, we evaluated whether colitis in IL-2Rα^−/−^ mice is amenable to assessment via USMI. The IL-2/IL-2Rα signaling pathway is crucial for the development and expansion of the CD4+/CD25+ T regulatory (T_reg_) cells that down-regulate T cell proliferation/activation. The absence of T_reg_ cells can result in tissue inflammation, severe anemia, and other immune abnormalities, which may lead to early mortality [26,31,32]. It has been shown that inflammatory phenotypes (such as colitis) are similar in IL-2^−/−^ and IL-2Rα^−/−^ mice [23,24]. Our results show that IL-2Rα^−/−^ mice developed spontaneous chronic colitis with 100% penetrance from proximal through the distal colon, with both sexes affected equally between 6–14 weeks of age and presented sustained colitis with a human IBD-like remission-flare pattern as shown on fluctuating USMI signals between 6–30 weeks. In addition, the relatively low mortality of IL-2Rα^−/−^ mice through 30 weeks is suitable for serial USMI exams.

IL-2Rα^−/−^ mice develop normally until 6 weeks of age [23,24,26,27]. Afterwards, colitis spontaneously develops in IL-2Rα^−/−^ mice housed in conventional environment, starting between 6 and 15 weeks of age, as reported previously [24,33]. The onset of colitis between 6–14 weeks in the current study is in agreement with previous studies [24,33]. To determine the sex and locational difference in colitis, we always scanned the proximal, middle, and distal colon in both male and female IL-2Rα^−/−^ mice. We did not find any significant differences either between proximal, middle. and distal colon, or between male and female mice between 6–30 weeks of age. Multiple bowel segments are usually involved in the IBD of patients. We imaged the three locations in the current study. Since there was no significant difference between proximal, middle, and distal colon, the imaging signal intensity in one location could reflect the inflammatory status of the entire colon. Furthermore, the mortality rate of 26.8% until 30 weeks in the current study was similar to the previous report [24]. The relatively long-time window (6–30 weeks) and acceptable mortality of the IL-2Rα^−/−^ mice with colitis provide ample room for long-term monitoring of anti-inflammatory treatment(s).

An interesting finding in the current study was the fluctuating P- and E-selectin targeted USMI signals of colitis in IL-2Rα^−/−^ mice between 6–30 weeks. Although some other factors may also contribute to the observed fluctuating USMI signals, such as slightly different amounts of MB_Selecin_ injected at each time point and inaccurate localization of the same colonic location for multiple follow-up imaging acquisition at each time point, the fluctuating USMI signals might suggest a remission-flare pattern of colitis in these animals. The chronic course of human IBD is variable and characterized by alternating phases of remission and flare, which vary in duration and frequency. An IBD flare may present with objective symptoms and signs such as abdominal pain or change in bowel habitats, which are not always in accordance with disease activity. Endoscopy with biopsy remains the gold standard for diagnosing IBD disease activity [34,35]. However, this is an invasive procedure with sedation, and thus not tolerated well in all patients. It has been confirmed that P- and E-selectin expression is substantially upregulated in active (flare) IBD than in the quiescent status (remission) and can be used as a quantitative index for IBD flares [14,15,16]. In the current study, the fluctuating P- and E-selectin targeted USMI signals of colitis might reflect the remission-flare pattern of sustained chronic colitis in IL-2Rα^−/−^ mice, which might resemble the phenotype of human IBD. It is noteworthy that the duration and frequency of remission flare-like fluctuating USMI signals vary in each mouse. P- and E-selectin targeted USMI can be performed in multiple bowel segments after one injection of MBs, and thus can be used as a non-invasive quantitative imaging tool to evaluate the extent and degree of IBD flare.

Chronic inflammatory changes were observed on H&E staining of IL-2Rα^−/−^ mice from weeks 14 to 30. Furthermore, overexpression of P- and E-selectin was observed on immunofluorescence staining in IL-2Rα^−/−^ mice at multiple time points when the low to high USMI signals were detected, but not observed in the IL-2Rα^−/−^ mice, where only background USMI signals were observed. It has been shown that in vivo dual-selectin targeted USMI signals correlate with ex vivo overexpression of P- and E-selectin [17,19,21], and overexpression of P- and E-selectin is the indicator of active IBD [14,15,16]. Therefore, dual-selectin targeted USMI signals could be used to monitor the remission-flare phases of IBD non-invasively.

We also acknowledge the following study limitations. Firstly, we did not perform three-dimensional USMI in the current study. To utilize the prolonged window of MB attachment to P- and E-selectin on vascular endothelial cells, we always performed imaging acquisitions of three colonic locations after one injection of MBs in order to combat the sampling limitations of two-dimensional USMI. With the technical advancement of volume transducers in the future, three-dimensional USMI will enable the overall evaluation of the entire mouse colon in one imaging acquisition. Secondly, we evaluated dual-selectin-targeted USMI only in colitis model of IL-2Rα^−/−^ mice. In future studies, other IBD models such as SAMP model of terminal ileitis, and other inflammatory molecular markers such as MAdCAM-1 should be explored as well to further validate the role of USMI in chronic mouse models of IBD. Lastly, the quantification of the USMI signal from attached MB_selectin_ was based on the difference between attached and freely circulating MB_selectin_. High mechanical index US pulses were used to destroy the attached MB_selectin_ in the FOV in the current study. When USMI is translated to the clinic, imaging protocols without using destructive pulses are preferred [36]. Hence, we will work on developing an algorithm to measure mobile and stationary MBs without applying destruction pulses as a strategy for quantitative USMI.

In conclusion, our results show that spontaneous chronic colitis in IL-2Rα^−/−^ mice is independent of sex and locational differences in the colon and could be detected between 6–30 weeks of age using P- and E-selectin targeted ultrasound molecular imaging. The fluctuating imaging signals in IL-2Rα^−/−^ mice might recapitulate the remission-flare pattern of human IBD. Thus, the chronic colitis model in IL-2Rα^−/−^ mice is suitable for the long-term assessment of treatment response with dual-selectin targeted ultrasound molecular imaging.

## Figures and Tables

**Table 1:** Mortality, frequency and onset of colitis in IL-2Rα^−/−^ mice.

Mortality	
IL-2Rα^−/−^ mice < 6 weeks	4.9% (2/41)
IL-2Rα^−/−^ mice between 6–30 weeks	26.8% (11/41)
Number of mice imaged	
Wild-type mice	6
Male	3
Female	3
IL-2Rα^−/−^ mice	39
Male	21
Female	18
**Number of USMI acquisitions and mice with positive USMI signals**	
Total USMI acquisitions of IL-2Rα^−/−^ mice	387
Male	201
Female	186
Total positive acquisitions with low-high USMI signals	42.9% (166/387)
Male	42.3% (85/201)
Female	43.5% (81/186)
Mice with at least one positive USMI imaging acquisition	100% (39/39)
Male	100% (21/21)
Female	100% (18/18)
**Onset of colitis**	Weeks
Earliest	6
Latest	14
Mean	10.5
Median	11

## Data Availability

The data presented in this study are available from the corresponding author upon request.

## Abbreviations

a.u.	arbitrary unit
CEUS	contrast-enhanced ultrasound
FOV	field of view
H&E	hematoxylin and eosin
IBD	inflammatory bowel disease
IL-2Rα^−/−^	interleukin 2 receptor α deficient
MBSelectin	dual P- and E-selectin targeted microbubbles
PBS	phosphate buffered saline
PFA	paraformaldehyde
OCT	optimal cutting temperature compound
ROI	region of interest
Treg	T regulatory
USMI	ultrasound molecular imaging
